# Response to GH Treatment After Radiation Therapy Depends on Location of Irradiation

**DOI:** 10.1210/clinem/dgaa478

**Published:** 2020-07-24

**Authors:** Susan R Rose, Martin Carlsson, Adda Grimberg, Ferah Aydin, Assunta Albanese, Anita C S Hokken-Koelega, Cecilia Camacho-Hubner

**Affiliations:** 1 Pediatric Endocrinology and Metabolism, Cincinnati Children’s Hospital Medical Center, University of Cincinnati College of Medicine, Cincinnati, Ohio; 2 Pfizer Inc., Endocrine Care, New York, New York; 3 Perelman School of Medicine, Univ. of Pennsylvania, Children’s Hospital of Philadelphia, Philadelphia, Pennsylvania; 4 Pfizer Health AB, Endocrine Care, Sollentuna, Sweden; 5 St George’s University Hospitals NHS Foundation Trust, London, UK; 6 Dutch Growth Research Foundation, Rotterdam, The Netherlands; 7 Erasmus University Medical Center, Sophia’s Children’s Hospital, Department of Pediatrics, Division of Endocrinology, Rotterdam, The Netherlands

**Keywords:** growth hormone therapy, growth hormone deficiency, pediatric, cancer survivors, dose response, radiation therapy

## Abstract

**Objectives:**

Cancer survivors with GH deficiency (GHD) receive GH therapy (GHT) after 1+ year observation to ensure stable tumor status/resolution.

**Hypothesis:**

Radiation therapy (RT) to brain, spine, or extremities alters growth response to GHT.

**Aim:**

Identify differences in growth response to GHT according to type/location of RT.

**Methods:**

The Pfizer International Growth Database was searched for cancer survivors on GHT for ≥5 years. Patient data, grouped by tumor type, were analyzed for therapy (surgery, chemotherapy, RT of the focal central nervous system, cranial, craniospinal, or total body irradiation [TBI] as part of bone marrow transplantation), sex, peak stimulated GH, age at GHT start, and duration from RT to GHT start. Kruskal-Wallis test and quantile regression modeling were performed.

**Results:**

Of 1149 GHD survivors on GHT for ≥5 years (male 733; median age 8.4 years; GH peak 2.8 ng/mL), 431 had craniopharyngioma (251, cranial RT), 224 medulloblastoma (craniospinal RT), 134 leukemia (72 TBI), and 360 other tumors. Median age differed by tumor group (*P* < 0.001). Five-year delta height SD score (SDS) (5-year ∆HtSDS; median [10th-90th percentile]) was greatest for craniopharyngioma, 1.6 (0.3-3.0); for medulloblastoma, 5-year ∆HtSDS 0.9 (0.0-1.9); for leukemia 5-year ∆HtSDS, after TBI (0.3, 0-0.7) versus without RT (0.5, 0-0.9), direct comparison *P* < 0.001. Adverse events included 40 treatment-related, but none unexpected.

**Conclusions:**

TBI for leukemia had significant impact on growth response to GHT. Medulloblastoma survivors had intermediate GHT response, whereas craniopharyngioma cranial RT did not alter GHT response. Both craniospinal and epiphyseal irradiation negatively affect growth response to GH therapy compared with only cranial RT or no RT.

GH deficiency is common after radiation therapy (RT) for treatment of childhood cancer (1-3). RT for childhood cancer may include cranial or craniospinal irradiation for brain tumor, nasopharyngeal tumor, orbital tumor, or central nervous system leukemia; or total body irradiation (TBI) as preparation for bone marrow transplantation. GH deficiency (GHD) is often the only pituitary hormone deficiency after low-dose cranial irradiation (18-24 Gy) (4, 5). Higher RT doses (≥30 Gy) may lead to multiple pituitary hormone deficiencies (deficiencies of GH, TSH, ACTH, and gonadotropins) (5-15). Risk, severity, and timing of onset of GHD are affected by the RT dose to the hypothalamus, not the pituitary (16-19). The risk of GHD after irradiation also increases with age younger than 10 years at time of tumor treatment (20, 21). Precocious puberty or rapid progression of puberty can also occur after cranial RT >18 Gy, with female sex and younger age at initial RT being the most important risk factors (22).

Of note, recommendations for GH replacement for GHD (based on expert opinion) are to wait 1 year or longer after completion of cancer therapy with no tumor growth before initiating GH therapy to ensure stable status/ resolution of tumor (3, 23, 24). GH treatment (GHT) can be delayed longer depending on individual patient circumstances. GHT is considered safe in patients after central nervous system (CNS) tumors (25, 26) and is not associated with an increased risk of tumor recurrence or secondary tumors (27, 28). However, GHT should be considered only cautiously in persons with GHD after multiple recurrences, metastases, highly malignant tumors, or genetic predisposition to cancer (29, 30). Safety data have enabled earlier initiation of GHT to lengthen the duration of therapy. Suppression of precocious, early, or rapid puberty may additionally increase adult height (31-34).

Tumor therapies (chemotherapy, RT) may affect subsequent growth of the skeleton (35-37). For instance, TBI is known to disrupt the epiphyses and reduce height potential in cancer survivors (36-38). Several studies have shown poor growth after fractionated RT (cranial, craniospinal, TBI), regardless of whether children developed GHD (39-44). It appears that GHT for GHD after craniopharyngioma increases adult height (45). Because irradiation damages both the epiphyses and the bone matrix, the skeleton may not demonstrate the growth response to GHT that is expected in children with idiopathic GHD (46).

We hypothesized that RT to brain, spine, or extremities alters linear growth response to GHT. We sought to assess the relative effects on growth response to GHT of cranial irradiation versus craniospinal irradiation versus RT to all growth centers (as in TBI) in a large group of childhood cancer survivors. The aim was to identify differences in growth response to GHT according to type and location of RT. We also reviewed the occurrence of adverse events in these cancer survivors during GHT.

## Patients and Methods

The Pfizer International Growth Database (KIGS) was searched for cancer survivors who had received GHT for at least 5 years. The 1149 cancer survivors were grouped by tumor type and data were analyzed for tumor therapy (surgery, chemotherapy, RT [focal CNS, whole brain, craniospinal, or TBI as part of bone marrow transplantation]), sex, peak stimulated GH level, age at GHT start, and duration from RT to GHT start. Details of imaging, specific chemotherapeutic agents, and irradiation dosimetry administered were not available in the database. Within tumor diagnosis, patients were grouped by presence or absence of RT (Table 1).

**Table 1.** **Characteristics of Patients With GH Deficiency in the KIGS Database who Have History of Childhood Cancer (n** = **1149)**

Male	733	64%
Median age of GHT start (y)	8.4	5.1, 11.6
Median peak GH (ng/mL)	2.8	0.5, 9.5
Median duration GHT (y)	7.5	5.5, 11.0
Median GH dose (mg/kg/wk)	0.18	0.12, 0.26
Tumor groups		
Craniopharyngioma total	431	38%
Cranial RT	137	32%
No RT	268	62%
Other/unknown	26	6%
Leukemia total	134	12%
TBI	71	53%
No TBI	52	39%
Other/unknown	11	8%
Medulloblastoma total	224	19%
Craniospinal RT	189	84%
Other/unknown	35	16%
Other tumor^*a*^	360	31%

Median and 10th, 90th percentiles are presented for continuous variables and Ns and % are for categorical variables.

Abbreviations: GHT, GH therapy; RT, radiation therapy; TBI, total body irradiation.

“Other” tumor group and unknown RT groups are excluded from analysis.

^*a*^Other tumor diagnoses: germinoma (dysgerminoma, pinealoma) 65; tumor of the pituitary/hypothalamic area 50; astrocytoma 41; ependymoma 35; glioma 35, solid tumor 27; nasopharyngeal tumor 23; lymphoma other 7; Hamartoma 6; non-Hodgkin lymphoma 4; other cranial tumors 67.

Children within each tumor group were categorized further as prepubertal versus pubertal at GHT start, based on testicular volume >3 mL or Tanner stage for breast development ≥2 as reported by the investigator to the KIGS database.

## Statistical Methods

Height was expressed in SD score (SDS) units from the mean for age and sex (HtSDS). Likewise, change in height was expressed as delta HtSDS (∆HtSDS).

A stepwise regression model was used to identify covariates for inclusion in a quantile regression model of HtSDS at year 5 and ∆HtSDS at years 1 and 5 as the dependent variables. The independent covariates were the background and start of GH variables listed in Table 2. Backwards stepwise variable selection was used with cutoff values of *P* < 0.05 for entry and retention.

**Table 2.** Characteristics of Cancer Survivors With GH Deficiency in the KIGS Database, According to Tumor Subgroup

	Craniopharyngioma (RT)	Craniopharyngioma (No RT)	Medulloblastoma	Leukemia (TBI)	Leukemia (No TBI)	*P* Value^*a*^
Background						
N, male %	**N = 139,** 56%	**N = 270,** 59%	**N = 224,** 75%	**N = 72,** 62%	**N = 52,** 75%	<0.001
Birth weight, SDS	-0.2 (-1.5, 1.1)	-0.2 (-1.5, 1.2)	0.0 (-1.3, 1.1)	0.1 (-1.5, 1.4)	-0.1 (-1.2, 1.1)	0.487
Mid-parental height SDS	-0.5 (-2.0, 0.8)	-0.3 (-1.9, 1.1)	-0.2 (-1.5, 1.0)	-0.2 (-1.9, 1.0)	-0.9 (-2.2, 0.8)	0.004
Maximum GH peak (ng/mL)	1.5 (0.3, 4.6)	1.2 (0.2, 4.9)	4.5 (1.6, 13.0)	7.3 (2.2, 15.0)	6.0 (1.4, 14.1)	<0.001
IGF-I SDS	-2.8 (-4.1, -1.3)	-2.7 (-3.8, -1.1)	-2.2 (-3.3, -0.8)	-1.2 (-4.4, 1.4)	-2.8 (-4.8, -2.6)	0.012
Age at tumor diagnosis, y	7.9 (4.5, 11.9)	7.3 (2.9, 11.6)	6.7 (3.6, 10.3)	5.3 (1.9, 10.8)	8.6 (2.4, 11.5)	<0.001
Age at starting cranial irradiation, y^*b*^	6.6 (4.6, 11.5) N = 41		5.4 (3.0, 8.0) N = 83	-		
Age at starting TBI, y^*b*^	-	-	-	4.7 (2.3, 7.8) N = 43	-	
Age at surgery, y^*b*^	-	6.8 (2.4, 10.1) N = 100	-	-	-	
Age at starting chemotherapy, y^*b*^	-	-	-	-	2.6 (0.7, 5.8) N = 15	
**At start of GH therapy**						
Chronological age, y	8.3 (5.1, 11.8)	7.9 (3.6, 11.8)	8.0 (5.6, 10.6)	9.5 (6.5, 11.5)	10.0 (7.1, 11.7)	<0.001
Height SDS, Prader	-2.4 (-3.8, -0.3)	-2.2 (-4.1, -0.4)	-2.0 (-3.6, -0.8)	-2.1 (-3.2, -1.0)	-2.2 (-3.5, -0.9)	0.528
Height - MPH SDS, Prader	-1.9 (-3.7, 0.1)	-1.8 (-3.6, -0.1)	-1.7 (-3.2, -0.6)	-1.6 (-3.1, -0.7)	-0.9 (-2.8, 0.5)	0.108
Weight SDS	-0.3 (-2.4, 1.6)	-0.4 (-2.2, 2.2)	-0.8 (-2.2, 0.5)	-1.2 (-2.3, 0.7)	-1.0 (-2.2, 0.8)	<0.001
BMI SDS, CDC/NCHS	1.2 (-0.5, 2.7)	1.2 (-0.8, 3.3)	0.4 (-1.0, 1.6)	-0.1 (-1.4, 1.7)	0.4 (-1.1, 1.8)	<0.001
GH dose, mg/kg/wk	0.17 (0.10, 0.25)	0.18 (0.12, 0.25)	0.20 (0.14, 0.27)	0.22 (0.17, 0.31)	0.18 (0.12, 0.27)	<0.001
**At latest reported clinic visit**						
Age at start of puberty^*b*^	13.5 (11.0, 16.3)	13,3 (11.3, 15.7)	11.5 (9.6, 13.8)	12.4 (11.2, 14.9)	12.1 (10.4, 14.7)	<0.001
Duration on GHT, y	8.1 (5.4, 12.1)	8.4 (5.5, 12.5)	7.4 (5.7, 10.8)	6.9 (5.3, 9.5)	6.5 (5.4, 9.8)	0.002
Percent in puberty after 5 y of GHT^*b*^	48%	44%	84%	85%	86%	<0.001

Top panel shows background; middle panel, at start of GHT; and bottom panel, at latest reported clinic visit. Values shown are median (10th, 90th percentiles).

Abbreviations: CDC/NCHS, Centers for Disease Control and Prevention/National Center for Health Statistics; GHT, GH therapy; IGF-I, insulin-like growth factor I; RT, radiation therapy; TBI, total body irradiation; SDS, SD score.

^*a*^Kruskal-Wallis test was applied to the continuous background variables, and χ ^2^ test to the binary variables.

^*b*^Based on genital stage (male) testicular volume >3 mL and breast stage ≥2 (female) reported by the investigator at GH start on the case report form.

*Tumor treatment ages were not available for all patients.

Quantile regression (47) was used to estimate the conditional median of height SDS at year 5 and ∆HtSDS at years 1 and 5, including the significant covariates identified in the stepwise model (Table 3). The quantile regression technique was chosen for the analyses because of the non-normal distributions of the data as tested with the Shapiro-Wilk test, invalidating assumption for ordinary least-squares regression. Quantile regression allows estimation and inference related to the median without making any distributional assumptions, in contrast to least-squares methods that rely on a normality assumption. The skewness of the height SDS variables may reflect meaningful heterogeneity. Kruskal-Wallis test was applied to the continuous background and start of GH variables and χ ^2^ test to the binary variables.

**Table 3.** Quantile Regression of 1- and 5-Year Change in Height SDS (∆HtSDS) and Height in SD Units From the Mean for Age (HtSDS) at 5 Years of GH Therapy, for Childhood Cancer Survivors With and Without Irradiation in GH-Treated Cancer Survivors With GH Deficiency Entered in the KIGS Database, Quantile 0.50 (median)

Exploratory Variable	1-Year ΔHtSDS		5-Year ΔHtSDS		HtSDS at 5 Years of GH	
	Estimate	*P* Value	Estimate	*P* Value	Estimate	*P* Value
Craniopharyngioma (RT)	0.75	<0.0001	2.24	<0.0001	-0.17	0.47
Craniopharyngioma (no RT)	0.80	<0.0001	2.25	<0.0001	-0.23	0.32
Leukemia (TBI)	0.27	0.015	0.90	0.0004	-1.74	<0.001
Leukemia (no TBI)	0.50	0.0001	1.69	<0.0001	-0.77	0.002
Medulloblastoma	0.47	<0.0001	1.48	<0.0001	-1.02	<0.001
Craniopharyngioma RT vs craniopharyngioma no RT	-0.05	0.47	-0.01	0.96	0.05	0.68
Craniopharyngioma RT vs leukemia (TBI)	0.48	<0.0001	1.34	<0.0001	1.56	<0.001
Craniopharyngioma RT vs leukemia (no TBI)	0.25	0.012	0.56	0.0004	0.59	<0.001
Craniopharyngioma RT vs medulloblastoma	0.28	<0.0001	0.76	<0.0001	0.85	<0.001
Craniopharyngioma no RT vs leukemia (TBI)	0.53	<0.0001	1.35	<0.0001	1.51	<0.001
Craniopharyngioma no RT vs leukemia (no TBI)	0.30	0.003	0.56	0.0004	0.54	<0.001
Craniopharyngioma no RT vs medulloblastoma	0.33	<0.0001	0.76	<0.0001	0.79	<0.001
Leukemia (TBI) vs leukemia (no TBI)	-0.23	0.011	-0.79	<0.0001	-0.97	<0.001
Leukemia (TBI) vs medulloblastoma	-0.20	<.0001	-0.58	<0.0001	-0.72	<0.001
Leukemia (no TBI) vs medulloblastoma	0.03	0.72	0.21	0.15	0.25	0.018
In puberty	0.57	<.0001	1.84	<0.0001	-0.56	0.019
Prepubertal	0.55	<.0001	1.58	<0.0001	-1.01	<0.001
In puberty vs prepubertal	0.02	0.70	0.26	0.024	0.45	<0.001
Age at GHT start (y)	-0.04	0.0003	-0.07	<0.0001	-0.06	0.002
Weight (SDS) at GHT start	0.04	0.003	0.07	0.39	NA	
Dose at GHT start (mg/kg/wk)	1.56	<0.0001	1.68	0.014	2.36	<0.001

Values shown are parameter estimates and *P* values from quantile regression at the 50th percentile.

Abbreviations: GHT, GH therapy; RT, radiotherapy; SDS, SD score; TBI, total body irradiation.

All statistical tests were carried out at the 2-sided significance level of 5%. Statistical analyses were performed using SAS, version 9.4 (SAS Institute Inc., Cary, NC) using the QUANTREG procedure. The NPAR1WAY and FREQ procedures were applied for univariate testing of the background and start of GH variables presented in Table 2.

## Results

### Patient characteristics

Of 1149 cancer survivors with GHD (male 733; median age 8.4 years; median GH peak 2.8 ng/mL) treated with GH in KIGS, 431 had craniopharyngioma (251 with cranial RT), 224 had medulloblastoma (all with craniospinal RT), and 134 had leukemia (72 with TBI) (Tables 1 and 2). (The remaining survivors in each category did not receive any RT.) The other 360 patients were diagnosed with a variety of other tumors, which were treated with diverse regimens as clinically indicated for each of the different tumors. For clarity, given the diversity of their tumor therapies, their growth response to subsequent GHT was not included in this analysis. (A list of their diagnoses is shown as a footnote to Table 1.) No data were available in the database regarding adequacy of other hormone replacement (i.e., thyroid, hydrocortisone, vasopressin, or sex steroids).

Background characteristics according to tumor group are presented in Table 2 (top) including univariate testing of the variables. Table 2 (bottom) shows characteristics according to tumor group at start of GHT and at last reported clinic visit. In the quantile regression model, response to GHT within tumor groups was associated with HtSDS at 5 years after controlling for puberty status, age at tumor diagnosis, age at start of GH, mid-parental height SDS, HtSDS at start of GH, body mass index (BMI) SDS at GHT start, and dose at GHT start (mg/kg/week).

### Height achieved during GHT according to tumor group

**Figure 1.**
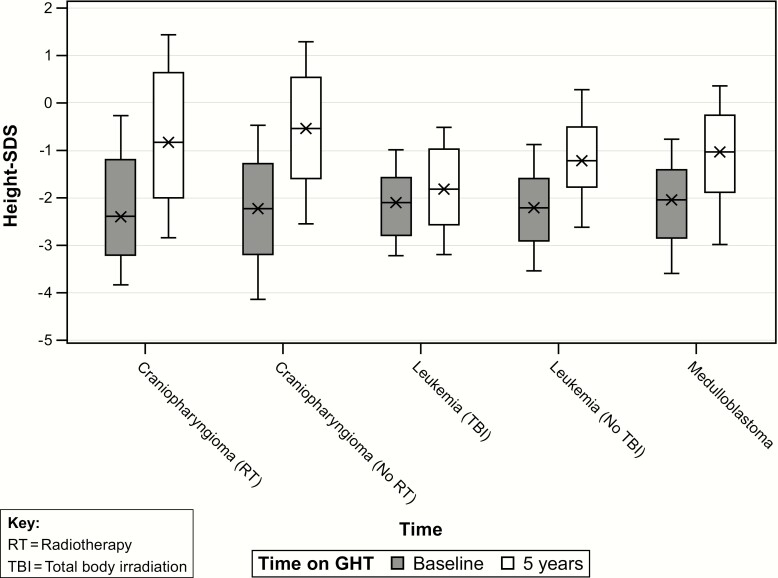
Height SD from the mean (HtSDS) at baseline before GH therapy (GHT) and after 5 years of GHT in GH-deficient childhood cancer survivors, according to tumor subgroup. SDS, SD score.

Craniopharyngioma (RT) was associated with the tallest 5-year HtSDS (median 5-year HtSDS estimate = -0.17) compared with the other tumor groups (*P* < 0.05), except for the craniopharyngioma (no RT) group (median estimate = -0.23, *P* = NS). The median 5-year HtSDS for the leukemia (TBI) was significantly lower than the other tumor groups (median estimate = -1.74, *P* < 0.05) (Fig. 1)

Factors significantly associated with taller 5-year HtSDS included dose at start of GH, HtSDS at start of GH, mid-parental HtSDS, and BMI SDS at GH start. Age at tumor diagnosis and age at GH start were negatively associated with 5-year HtSDS (*P* < 0.05). Results are displayed in Table 3 (top).

### Effects of initial pubertal status on HtSDS

**Figure 2.**
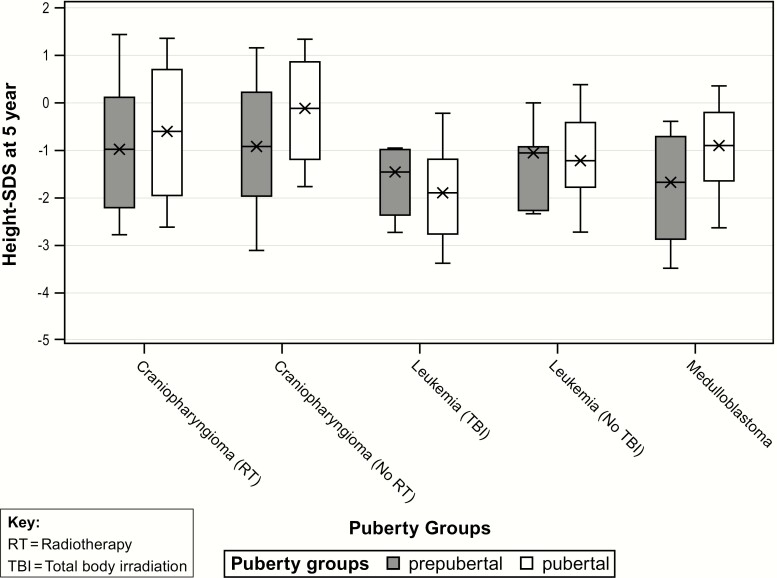
Height SD from the mean (HtSDS) at 5 years of GH therapy (GHT) in prepubertal compared with pubertal GH-deficient childhood cancer survivors, according to tumor subgroup. SDS, SD score.

Overall, patients who were pubertal at start of GHT had a significantly taller 5-year HtSDS compared with prepubertal patients, with an estimated difference of median 5-year HtSDS effect equal to 0.45 (*P* < 0.0001) (Table 4 bottom, Fig. 2). This was in spite of the pubertal and prepubertal groups having a similar HtSDS at baseline. Results for each tumor group according to pubertal status at start of GHT are illustrated in Fig. 2. Interestingly, there was a significant interaction effect between tumor group and puberty status: patients in the prepubertal leukemia groups had a taller median 5-year HtSDS compared with pubertal patients. An opposite effect was observed for the 3 other tumor groups in which prepubertal patients had a lower 5-year HtSDS than did the pubertal patients. The median age at GHT start for pubertal patients was 9.3 years versus 6.4 years for prepubertal patients, *P* < 0.001.

**Table 4.** Comparison of 1- and 5-Year Height in SDS from the Mean for Age (HtSDS) and Change in Height SDS (∆HtSDS) During GH Therapy, for Childhood Cancer Survivors With and Without Irradiation

Tumor Groups: All Patients	Median Delta Height SDS (10th, 90th percentiles)			
	HtSDS at Start of GH	1-Year ∆HtSDS	5-Year ∆HtSDS	HtSDS at 5 Years of GH
Craniopharyngioma (RT + no RT)	-2.3 (-3.9, -0.4)	0.8 (0.1, 1.5)	1.6 (0.3, 3.0)	-0.6 (-2.6, 1.4)
Leukemia (TBI)	-2.1 (-3.2, -1.0)	0.3 (0.0, 0.7)	0.4 (-0.6, 1.3)	-1.8 (-3.2, -0.5)
Leukemia (no TBI)	-2.2 (-3.5, -0.9)	0.5 (0.0, 0.9)	1.0 (0.2, 1.9)	-1.2 (-2.6, 0.3)
Medulloblastoma (craniospinal RT)	-2.0 (-3.6, -0.8)	0.5 (0.1, 0.9)	0.9 (0.0, 1.9)	-1.0 (-3.0, 0.4)
**Tumor Groups: Prepubertal Patients**				
**Craniopharyngioma (RT + no RT)**	**-2.5** ^***a***^ **(-4.3, -0.7)**	**0.8** **(0.1, 1.7)**	**1.6** **(0.3, 3.3)**	**-0.8** ^***a***^ **(-2.9, 1.4)**
Leukemia (TBI)	-2.0 (-3.2, -1.2)	0.3 (0.0, 0.9)	0.4 (0.0, 1.7)	-1.4 (-2.7, -0.5)
Leukemia (no TBI)	-2.2 (-3.2, -1.6)	0.5 (0.1, 0.9)	1.1 (-0.7, 1.8)	-1.1 (-2.3, 0)
Medulloblastoma (craniospinal RT)	-2.4 (-4.0, -1.4)	0.5 (-0.1, 0.8)	0.9 (0.1, 1.7)	-1.6 (-3.5, -0.2)

Top panel includes all patients; bottom panel includes only patients who were prepubertal at start of GHT.

Abbreviations: RT, radiation therapy; TBI, total body irradiation; SDS, SD score.

^*a*^Data are presented as median delta height SD score (10th, 90th percentiles).

### Change in HtSDS during GH therapy according to tumor group

For the change in HtSDS (∆HtSDS) quantile regression models, response to GHT was significantly associated with tumor group and characteristics at GHT start (pubertal status, age, weight SDS, and initial GH dose).

**Figure 3.**
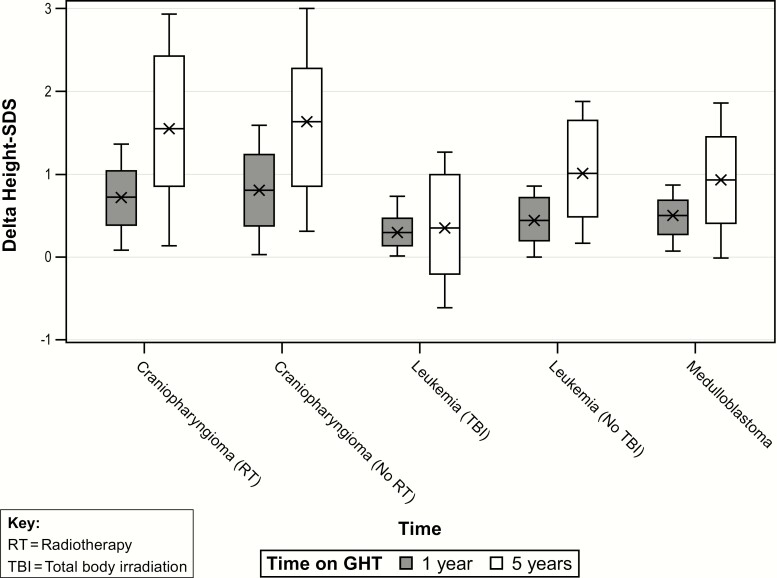
Change in height SD from mean baseline HtSDS (∆HtSDS) during GH therapy (GHT) in GH-deficient childhood cancer survivors at 1 and 5 years of GHT, according to tumor subgroup. SDS, SD score.

One- and 5-year median changes in height SDS (∆HtSDS) were greatest among tumor subgroups for craniopharyngioma, median (10th-90th percentile) 0.8 (0.1-1.5) and 1.6 (0.3-3.0), without cranial RT (1- and 5-year values 0.8 [0-1.8], 1.6 [0.3-3.0]), and with cranial/focal RT (1- and 5-year values 0.7 [0.1-1.4], 1.5 [0.2-2.9], *P* = NS) (Table 4 top). Delta HtSDS effects at 5 years of GHT for craniopharyngioma (RT) and (no RT) were associated with largest response to GHT (median 5-year ∆HtSDS estimates 1.5, 1.6) compared with other tumor groups (*P* < 0.05) (Fig. 3).

For medulloblastoma, 1- and 5-year ∆HtSDS were 0.5 (0.1-0.9) and 0.9 (0.0-1.9), respectively. This response was decreased compared with craniopharyngioma both with and without cranial RT (adjusted *P* < 0.0001).

For leukemia, 1-year ∆HtSDS after TBI was 0.3 (0.0-0.7) compared with 0.5 (0.0-0.9) for leukemia without RT, adjusted *P* = 0.011. Five-year ∆HtSDS after TBI was only 0.4 (-0.6 to 1.3) compared with 1.0 (0.2-1.9) for leukemia without RT (direct comparison adjusted *P* < 0.0001). These responses were decreased compared with craniopharyngioma at 1 and 5 years (adjusted *P* < 0.005). The median ∆HtSDS at 5 years for the leukemia (TBI) group was significantly less than for the other tumor groups (median 5-year ∆HtSDS estimate = 0.40, *P* < 0.05). The comparisons of median ∆HtSDS at 1 and 5 years in the leukemia (no TBI) versus medulloblastoma groups were not significant.

Pubertal patients had a greater 5-year ∆HtSDS compared with prepubertal patients, with an estimated difference of median effects equal to 0.26 (*P* < 0.024).

As expected, given the shorter time duration, the median 1-year ∆HtSDS effects were smaller compared with 5 years, with similar significant tumor group comparisons (Fig. 3). There was no significant effect of puberty on ∆HtSDS at 1 year of GHT. Increased ∆HtSDS was observed with higher weight SDS, median ∆HtSDS equal to 0.04. Dose at start of GHT was significantly associated with 1-year ∆HtSDS. Older age at GH start was associated with significantly lower ∆HtSDS of -0.04 for each 1-year increment in age at GH start. Results for ∆HtSDS at years 1 and 5 are displayed in Table 4 (top).

Dose at start of GHT was significantly associated with an increased ∆HtSDS. Older age at GHT start was associated with a significantly lower ∆HtSDS of -0.07 for each 1-year increment in age at GHT start.

### Growth for prepubertal cancer survivors (Table 4 bottom)

One- and 5-years median HtSDS were greatest among tumor subgroups for craniopharyngioma, median (10th-90th percentile) 0.8 (0.1-1.5) and 1.6 (0.3-3.0). For medulloblastoma, 1- and 5-year ∆HtSDS were 0.5 (-0.1 to 0.8) and 0.9 (0.1-1.7), respectively. For leukemia, 1-year ∆HtSDS after TBI was 0.3 (0.0-0.7) compared with 0.5 (0.0-0.9) for leukemia without RT. Five-year ∆HtSDS after TBI 0.4 (-0.6 to 1.3) compared with 1.0 (0.2-1.9) for leukemia without RT (direct comparison *P* < 0.05).

### Safety/adverse events

There were no new serious adverse events (SAE) that have not been previously reported. Forty patients reported a treatment-related SAE (Table 5); none of the SAE were unexpected. Drug was withdrawn in 18 of the 40 patients who had treatment-related SAEs, mainly from those with craniopharyngioma recurrence. Dose was delayed in 9 while SAEs resolved. In the others, there was no change in GH therapy.

**Table 5.** Treatment-Related Serious Adverse Effect Reported in 40 Cancer Survivors Treated With GH for GH Deficiency

Category	Diagnosis	No. of Patients
General and metabolic	Fatigue	1
	Type 1 diabetes mellitus	2
	Type 2 diabetes mellitus	1
Musculoskeletal	Muscle spasm	1
	Scoliosis	3
	Hip epiphysiolysis	1
	Peripheral edema	1
Neurologic	Headaches	4
	Epilepsy	2
Tumor	PNET progression	1
	Recurrence of craniopharyngioma	20
	Recurrence of Ewing sarcoma	1
	Recurrence of optic glioma	3 (1 death)

Abbreviation: PNET, primative neuroectodermal tumor.

Overall, there were 1190 adverse events reported in 420 patients, with 325 considered as serious in 193 patients. Death was reported for 20 patients, of whom 4 deaths occurred after cessation of GHT (intracranial hemorrhage, suicide, T-cell lymphoma, and bone neoplasm) (Table 6).

**Table 6.** Cause of Death in GH-Treated Cancer Survivors With GH deficiency, According to Tumor Group

Primary Diagnosis	N (male, N)	RT (N)	Age at GH Start (y)^*a*^	GH Dose (mg/kg/wk)	Age at SAE (y)^*a*^	Cause of Death
**Craniopharyngioma**	6 (2)	3 C 3 none	7.1 (4.0-10.7)	0.14 (0.05-0.20)	16.1 (12.1-18.6)	Craniopharyngioma recurrence (n = 2) Intracranial hemorrhage (n = 1) Unknown (n = 3)
**Leukemia**	2 (2)	1 C and G 1 TBI	8.6 (7.6-9.5)	0.18 (0.13-0.22)	16.6 (13.9-19.3)	Oligodendroglioma Suicide
**Medulloblastoma**	4 (3)	1 C 3 CS	7.7 (3.9-8.8)	0.12 (0.05-0.19)	15.0 (11.6-16.8)	Tumor recurrence Colorectal cancer Pneumonia Sudden collapse
**Others**	8 (4)	4 C 1 CS 2 TBI 1 none	7.5 (4.5-11.4)	0.21 (0.10-0.23)	16.6 (10.6-22.9)	Tumor recurrence (n = 2) Brain stem syndrome Hepatocellular carcinoma T-cell lymphoma Myelodysplastic syndrome Bone neoplasm Epilepsy

Abbreviations: C, cranial radiation therapy; CS, craniospinal radiation therapy; G, gonadal radiation therapy; TBI, total body irradiation; SAE, serious adverse event.

^*a*^Median (10^th^-90^th^ percentiles)

### Treatment discontinuation

Of the entire group of 1149 patients, 656 reported discontinuation of GHT. The most frequent explanations for discontinuation included completion of linear growth (N = 341, 52%), followed by poor height velocity (154, 23.5%), adverse event (22, 3.4%), lost to follow-up (20, 3.0%), and other reasons (119, 18.1%). The majority of patients who discontinued in the “other reasons” category withdrew for the following reasons: decision made by patient or parent (N = 55), no funds for GH (N = 16), change of product (N = 8), nonadherence (N = 6), not responding to treatment (N = 6), patient moved (N = 5), study closure (N = 3), treatment trial (N = 2), closing center (N = 2), change of diagnosis (N = 1), and other unknown (N = 15). The median duration of GHT at time of discontinuation for all reasons was 7.7 years, ranging from 6.4 years for adverse event to 8.2 years for near-adult height reached.

## Discussion

The current study shows that at 1 and 5 years of GHT, leukemia survivors with GHD who had received TBI as part of preparation for bone marrow transplantation experienced the most significant impact of RT on their growth response to GHT compared with other tumor survivors with GHD. After medulloblastoma (typically treated with craniospinal RT), survivors had an intermediate growth response to GHT. After craniopharyngioma (treated either without RT or with cranial RT), survivors did not have reduced growth response to GHT. Thus, we observed that both craniospinal RT and general epiphyseal irradiation (TBI) were associated with restricted linear growth response during GH treatment for GHD compared with cranial RT only or no RT.

Several studies (that were smaller than the current one) have observed that vertebral growth slows after spinal irradiation or TBI, which are tumor therapies that contribute to short stature (35-37). Additional factors contributing to poor growth velocity in tumor survivors may include age at tumor therapy, hypothyroidism, sex, altered pubertal timing, GH insufficiency, secondary adrenal insufficiency, chronic unresolved illness, undernutrition, depression, and suppressive effects of chronic steroid therapy for graft-versus-host disease (41, 48). GH therapy after craniopharyngioma clearly increases adult height (45).

GHD in craniopharyngioma represents a probable direct effect of tumor burden as well as resulting from surgery in the hypothalamic-pituitary region. Many patients with craniopharyngioma demonstrated slowing of linear growth before any tumor therapy (49, 50). Tumor involvement of the hypothalamus may partially explain why the patient groups with craniopharyngioma receiving RT and without RT in the current study had similar baseline HtSDS and similar growth responses to GHT. GH therapy is often started in GHD craniopharyngioma survivors by 1 year after cranial surgery, before observing the effects of GHD on height.

In contrast, development of GHD after medulloblastoma or leukemia represents an effect of RT. In addition, start of GHT in GHD survivors of medulloblastoma or leukemia is usually delayed for more years after RT; thus, such patients may have significant growth deficit by the start of GHT.

Several studies have shown poor growth after fractionated RT (cranial, craniospinal, or TBI), regardless of whether children developed GHD (40-44). In addition, growth response to GHT is less after RT to epiphyseal growth centers (36). Irradiation damages both the epiphyses and the bony matrix; thus, the skeleton may not demonstrate the growth response to GHT that is expected in children with idiopathic GHD (46).

The observation that leukemia survivors with GHD who received no RT grew no better during GHT than medulloblastoma survivors (who had received craniospinal RT) suggests long-term effects on growth potential by other factors in leukemia therapy (such as steroids or chemotherapy). Several studies have observed absence of catch-up growth after completion of therapy in leukemia survivors who had received no RT. Lack of catch-up growth was associated with reduced adult height (51, 52). Specific causative factors were not identified; however, younger age at leukemia diagnosis and greater chemotherapy intensity were associated with shorter adult height (51). It is possible that GHT may not resolve all of the growth deficit in leukemia survivors.

It is unclear whether GHT can compensate for the effects of RT on epiphyseal growth. A comparison of cranial compared with craniospinal RT regarding growth response to GHT has been performed in small cohorts only (44, 53-57). In addition, comparisons within a common study have not been made of the effects of cranial and craniospinal RT to those of TBI on response to GHT, nor to the growth response to GH in children with idiopathic GHD without cancer history.

The strengths of the current analysis derive from the large number of patient observations in each tumor group and the at least 5-year duration of observed GHT response. The study is limited by not having access in the database to details of imaging, tumor treatment details, body proportions such as sitting height measurements, details of timing of puberty, and other pituitary hormone dysfunction(s) and their treatment. These limitations are inherent in an analysis from a registry, where not all details from each patient were entered. Details about body proportions would be particularly useful in evaluating response to GHT in medulloblastoma patients who are treated with craniospinal RT.

It is likely that the growth response to GHT in cancer survivors with GHD could be associated with the RT dose to the hypothalamus or affected by additional factors not available in the database (precise pubertal timing, other hormone replacements, bone maturation).

Confounders in the analysis of the growth response to GHT are age and variation in the onset and tempo of puberty. Children who are younger at the onset of GHT have a faster initial growth velocity in response to GH than do those who are older at onset of GHT. In addition, timing of onset of puberty is often earlier after cranial RT, and rate of progression of puberty is often faster after cranial RT than in children without history of RT (33). These factors were not consistently reported in the KIGS database and could potentially affect our results. However, we did not observe less growth achieved over 5 years in our patients who received cranial RT compared with those without RT history.

Cancer survivors who were pubertal at the start of GHT generally reached a taller HtSDS after 5 years of GHT than did those who were prepubertal at start of GHT. Most likely, this was related to the pubertal growth spurt augmenting the growth response to GHT. Patients treated for leukemia with bone marrow transplantation with TBI were the exception. Their 5-year change in HtSDS was less than the prepubertal leukemia TBI group, perhaps because TBI (irradiation to all epiphyses) blunted the pubertal growth spurt.

Guidelines from the Endocrine Society, the Children’s Oncology Group, and from transplant groups recommend that in childhood cancer survivors, monitoring (height, weight, BMI, and puberty) should be every 6 months until adult height is achieved, then yearly in individuals who are at risk of GHD (cranial irradiation >18 Gy) (3, 54, 58). Per Pediatric Endocrine Society guidelines, “For GH initiation after completion of tumor therapy with no evidence of ongoing tumor, a standard waiting period of 12 months to establish ‘successful therapy’ of the primary lesion is reasonable but can also be altered depending on individual patient circumstances” (23). Initial recommended dose of GHT in children with GHD is 0.16-0.24 mg/kg per week divided daily (22-35 μg/kg per day), administered subcutaneously, with individualization of subsequent dosing (23, 25). The GH doses in the current report were generally near the lower end of this dose range. We suggest that GH doses be adjusted to achieve target IGF-I in the mid-normal range in cancer survivors (59), and that screening for additional endocrinopathies should be performed at least annually after RT (20). Adverse effects of GHT are rare, occur soon after therapy initiation, and include benign intracranial hypertension, slipped capital femoral epiphysis, scoliosis progression, or carpal tunnel syndrome (23). If coexisting ACTH deficiency is present, cortisol therapy should be started before GH or thyroid therapies.

Long-term surveillance is mandatory for childhood CNS tumor survivors treated with GH (3, 23, 25). It is important for clinicians to give patient-families realistic expectations of response to GHT so they can make appropriate, well-informed risk-benefit decisions (and also help prevent loss of adherence to GHT from disappointment that height is not increasing as anticipated). Those with a history of spinal RT should be prepared for suboptimal height increases as well as disproportionate elongation of legs relative to trunk. Otherwise, parents may request escalation of GHT doses, which still will not overcome RT effects but may increase risk for potential side effects. If families have appropriate expectations regarding limitations of GHT, hopefully there will be less pressure to pursue these strategies.

Clearly, GHT is effective in increasing growth achieved after tumor therapy in cancer survivors who have GHD. That less growth response is observed after leukemia with no RT, spinal RT, or TBI than in children without these treatments does not contravene use of GHT. Timing of onset and rate of progression of puberty may alter potential height gain. Use of GnRH analog therapy may prolong the cancer survivor’s ability to respond to GHT (but may increase disproportion). The goal of GHT in these children is to assist cancer survivors in returning toward normal stature.

## Data Availability

The datasets generated during and/or analyzed during the current study are not publicly available but are available from the corresponding author on reasonable request.

## Abbreviations

BMI: body mass index
CNS: central nervous system
GHD: GH deficiency
GHT: GH therapy
KIGS: Pfizer International Growth Database
RT: radiation therapy
SAE: significant adverse event
SDS: SD score
TBI: total body irradiation

## Additional Information

***Disclosure Summary:*** A.G. and A.H.-K. were members of the KIGS Steering Committee and received consultancy fees from Pfizer. A.G. was a consultant for the Pediatric Endocrine Society GHD Knowledge Center, sponsored by Sandoz. M.C. and C.C.-H. are full-time employees of Pfizer and F.A. was a full-time employee of Pfizer at the time of this study. S.R.R. and A.A. have no conflicts of interest to declare.
